# Image analysis optimisation for carotenoid and anthocyanin content prediction in carrots: addressing colour parameter multicollinearity and genotypic diversity

**DOI:** 10.3389/fpls.2026.1713048

**Published:** 2026-05-20

**Authors:** Alma C. Verme, Mark Melchior, Jan H. van den Berg, Carmen M. Padilla-Díaz

**Affiliations:** Brightlands Future Farming Institute, Department of Plant Envirogenetics, Faculty of Science and Engineering, Maastricht University, Venlo, Netherlands

**Keywords:** antioxidants, CIELab, colorimetry, *Daucus carota*, imageJ, partial least squares (PLS) regression, pigment content, plant pigment

## Abstract

**Introduction:**

Colorimetric analysis of food using the CIELab/Ch colour space (i.e., from digital images of samples) is an accessible, non-destructive method for carotenoid and anthocyanin content prediction. Literature presents very well-fit, but rudimentary, models for pigment estimation (e.g., single/multiple linear regressions). However, standardised methods that statistically account for the high multicollinearity between CIELab/Ch colour parameters, varying light conditions and colour calibration, and samples with high genotypic variability are lacking.

**Methods:**

An image analysis optimisation was developed for the prediction of carotenoid and anthocyanin content of 16 carrot genotypes of different colours. Samples were photographed under six light conditions with a digital camera and image colour was calibrated before analysis with the CIELab/Ch colour space. Total pigment contents and individual carotenoid contents were analysed chemically via spectrophotometry and high-performance liquid chromatography, respectively. Partial least squares (PLS) regressions were used to assess the colour-pigment relationships to correct for high multicollinearity amongst the independent variables (CIELab/Ch colour parameters).

**Results/discussion:**

The PLS models achieved satisfactory accuracy for the prediction of total carotenoid content (*ca. R^2^* = 0.77) and total anthocyanin content (*ca. R^2^* = 0.81) under all light conditions. The two models are suggested as robust approaches to total pigment prediction with multi-dimensional colour spaces, varying light conditions, and for a sample group of high genotypic variability. The carrot samples proved to have very high genetic diversity within each cultivar, resulting in unsatisfactory models for prediction of individual carotenoids (*ca. R^2^* = 0.45) under the default light condition. However, all the results can be used to expand databases (towards artificial intelligence) and aid breeding programmes in search for higher concentrations of these interesting antioxidants for human health.

## 1 Introduction

Carotenoids and anthocyanins are major classes of pigments responsible for a wide range of colours in fruits and vegetables, from yellow, orange, and red to purple and blue. Carotenoids provide yellow-orange and some red hues, while anthocyanins present other shades of red and purple-blue (Amorim-Carrilho et al., 2014; Holme et al., 2021). Both pigment groups are potent antioxidants when consumed in the diet and act through various mechanisms, compiling them into a sturdy group of nutraceuticals. Additionally, vibrant colours stimulate appetite and digestion, and consumers have been found to directly associate the colour of fresh produce to its quality (Munsch et al., 1983; Pérez et al., 2022).

The antioxidant properties of carotenoids and anthocyanins result from their relatively unstable chemical structure, rendering the pigments excellent prooxidant-reducing agents. Carotenoids have been reported to quench excited states of oxygen through triplet energy transfer, and can scavenge peroxyl radicals given their lipophilicity (Stahl and Sies, 2003; Meléndez-Martínez et al., 2007). Similarly, the scavenging activity of anthocyanins occurs via hydrogen or electron transfers (Miguel, 2011). However, the chemical instability of carotenoids may occur due to light oxidation, and in anthocyanins due to changes in pH. High performance liquid chromatography (HPLC) and spectrophotometry, the current standards for biochemical pigment quantification, may be destructive of the pigments’ chemical structures, are laborious, and require expensive equipment (Ruiz et al., 2008; Itle and Kabelka, 2009). Therefore, thorough investigation and standardisation of colour-pigment relationships is instrumental to perform fast and accessible quantifications, which is of high interest to crop breeding programmes (Ruiz et al., 2008).

Colorimetric assessment has been proposed as a non-destructive and more accessible approach for pigment estimation in different kinds of foods, and/or as assistance to other quantification methods (Ruiz et al., 2008; Moresco et al., 2017; Bunluephan et al., 2023). Colorimetry is generally performed with tristimulus colorimeters or digital image analysis models (Meléndez-Martínez et al., 2003; Goñi and Salvadori, 2017; Moresco et al., 2017; Agarwal et al., 2021). The multi-dimensional colour space CIELab/Ch is widely used to objectively measure colour because it is device-independent and covers the complete spectrum of human vision (Phillips, 2023). The colour parameters include L* (lightness or amount of white), a* (redness on the green-to-red scale), and b* (yellowness on the blue-to-yellow scale). From these, chromaticity (C*; saturation or intensity) and hue angle (*h°*) can be calculated (Vieira et al., 2019; Phillips, 2023).

Currently, results amongst the literature on colorimetric pigment prediction are inconsistent and the field lacks a dependable standardised method. Colour calibration approaches in existing studies often do not account for the spectral properties of illumination or their interaction with camera sensor response (Agarwal et al., 2021; Kim and Van Iersel, 2023). Additionally, the influence of light intensity on pigment prediction has been assessed by some (Eoh et al., 2025), however no widely adopted protocol has been established to standardise image acquisition conditions, particularly with respect to lighting configuration (e.g., polarisation, angle, and intensity) and their influence on measured colour parameters. These factors are critical, as variations in the intensity, spectral composition, and geometry of illumination can significantly influence measured colour parameters. For instance, higher moisture content on the surface of samples was found to increase the refracted light registered by the sensor (or simply the human eye), thus increasing L* and C* values (Özkan et al., 2003).

Comparably, samples with higher pigment content have darker flesh colour (L* value decreases). The literature describes a strong negative relationship (*R^2^* = 0.61 – 0.98) between L* in samples and total carotenoid content (TCC), β-carotene, lycopene, lutein, and total anthocyanin content (TAC) individually in different kinds of foods (D’Souza et al., 1992; Takahata et al., 1993; Meléndez-Martínez et al., 2003; Montes et al., 2005; Simonne et al., 1993; Itle and Kabelka, 2009; Pandurangaiah et al., 2020; Bunluephan et al., 2023). Amongst the studies reviewed, only Fernandes Aquino et al. (2016) made explicit a lack of relationship between carotenoids and L* in different varieties of banana.

Parameter a* was found to be the best predictor of TCC in different kinds of processed baby foods by Dóka et al. (2014) (*R^2^* = 0.99) and in squash by Itle and Kabelka (2009) (*R^2^* = 0.83). High correlations are reported between a* and lycopene as well, in different foods (D’Souza et al., 1992; Hyman et al., 2004). Contrarily, Meléndez-Martínez et al. (2003) found that yellowness (b*) and C* were the highest correlated parameters with carotenoid content in orange juice (*R^2^* = 0.95 – 0.999), as they were for lutein estimation in squash according to Kim et al. (2012) and Itle and Kabelka (2009).

TAC, on the other hand, was also found to correlate highly with C* in Jaboticaba fruit according to Montes et al. (2005). However, Cuevas Montilla et al. (2011) found that increasing anthocyanin concentration in purple-black carrots was related with increased *h°* values instead. They reported increasing *h°* values as samples changed from more reddish hues towards black. Therefore, lower TAC (in reddish roots) also coincided with higher a* values (Cuevas Montilla et al., 2011). Unlike most literature, Munsch et al. (1983) showed that *all* colour parameters are required for carotenoid prediction given the multicollinearity of the parameters and their distinct influence on each other. Moyano et al. (2008) obtained models with significantly better fits when correlating TCC to L*a*b* (*R^2^* = 0.82 – 0.84) and L*C*h° (*R^2^* = 0.80 – 0.82) than to the individual colour parameters (*R^2^* = 0.24 – 0.64). The inconsistencies found between studies in the field imply that the use of rudimentary regression analyses should be critically evaluated by using more robust statistical methods, such as partial least squares (PLS), as suggested by some studies (Meléndez-Martínez et al., 2003; Han et al., 2008; Ruiz et al., 2008; Moresco et al., 2017). Additionally, Seroczynska et al. (2006) observed that despite the CIELab/Ch colour space proving a very strong method of TCC and β-carotene estimation in winter squash flowers, the estimation results were weak in the fruit flesh of the same plants - suggesting unreliability when extrapolating the models to different kinds of samples. A comprehensive overview of the crops, tissues, and food products included in this literature review is available in: https://doi.org/10.34894/OUURRH.

Carrots (*Daucus carota*, L.) may be rich in carotenoids and/or anthocyanins, with a wide possible gamut of phenotypic variety in root colour (cultivars ranging from white to purple/black) (Meléndez-Martínez et al., 2003; Surles et al., 2004). However, robust colorimetric research on such a group of samples is currently lacking. The TCC of orange carrot roots typically ranges between 8–25 mg/100 g fresh weight (FW), accounting for an excellent dietary contribution (Dóka et al., 2014). This composition is roughly 45 – 80% β-carotene, 10 – 20% α-carotene, and 1 – 2% lutein (Surles et al., 2004). Lycopene varies more amongst cultivars, with some containing up to 6% (Surles et al., 2004). Anthocyanins can accumulate in carrots in very high quantities, resulting in (dark) purple colours amongst the purple/black cultivars. The TAC range can broadly vary between 0.5–190 mg/100 g FW in these cultivars, some exhibiting anthocyanins in combination with other pigments (mainly β-carotene and α-carotene) (Holme et al., 2021; Pérez et al., 2022).

The purpose of this study is to develop an image analysis optimisation (IAO) to assess if total carotenoid, lutein, lycopene, α-carotene, β-carotene, and total anthocyanin contents of 16 carrot cultivars can be predicted from their root colour (ranging from white to purple). The novelty of this study is the use of six different light conditions, combined with colour calibration during the acquisition and analysis of the digital images. Importantly, PLS regressions are used as a robust statistical method that accounts for the high multicollinearity of the CIELab/Ch colour parameters (L*, a*, b*, C*, and *h°*), which is rarely accounted for in other research in the field.

## 2 Materials and methods

### 2.1 Plant material

**Figure 1:**
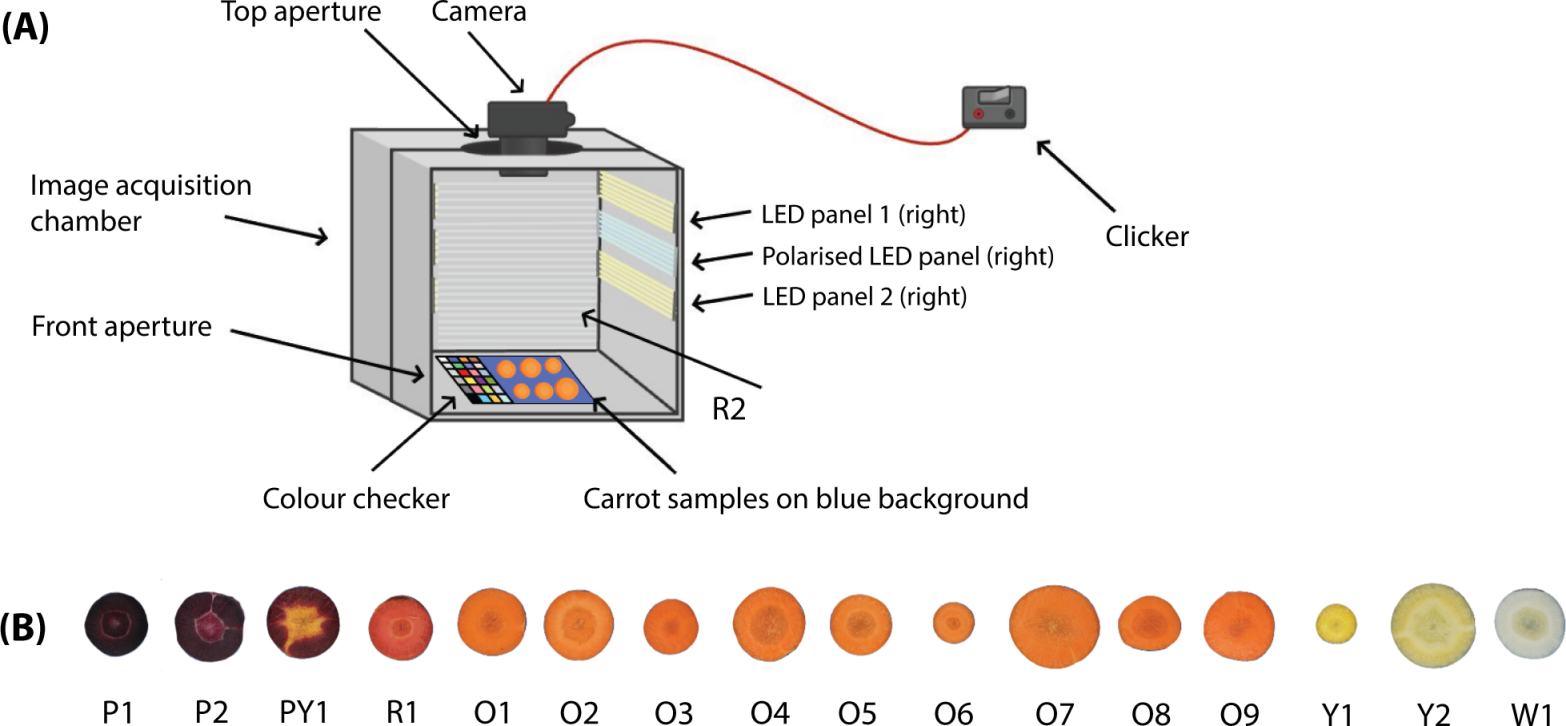
**(A)** Diagram of image acquisition chamber of the image analysis optimisation. Black photo box (60 *cm^3^*, represented in grey for visualisation purposes) with camera pointing towards carrot disk samples and colour checker (on blue background) from above. Photography supported by remote control clicker. Chamber equipped with six LED panels (only three visible; right). **(B)** Grouping of the selected cultivars based on root colour, illustrating the phenotypic variability: white (W1), yellow (Y1, Y2), orange (O1–O9), red (R1), purple (P1, P2), and purple with a yellow inner core (PY1).

16 carrot cultivars were grown in a field in Roggel, the Netherlands (51.26°N, 5.92°E) from 14 May to 30 October, 2023. The different cultivars were chosen to study an extensive phenotypic variability and may be grouped by colour: white (W1), yellow (Y1, Y2), orange (O1, O2, O3, O4, O5, O6, O7, O8, O9), red (R1), purple (P1, P2), and purple with a yellow inner core (PY1) (**Figure 1B**). The samples were all harvested 5 months after sowing, on the same day, and stored in black plastic bags at 4 °C to avoid exposure to light and the degradation of pigments until image acquisition (30 October, 2023) and pigments extraction (31 October, 2023) (Cinar, 2004).

### 2.2 Image acquisition

Image acquisition of the carrot samples was performed in a medium size lightbox (HPB-60XD Photo Studio 2021, HAVOX, Hong Kong, China) medium size lightbox, consisting of a cube chamber to exclude ambient light with 60 cm edges and apertures in the front (for access) and on the top (for the camera lens) (**Figure 1A**). The digital camera (EOS 90D, Canon, Tokyo, Japan) equipped with the lens (EF-S 17–55 mm f/2.8 IS USM, Canon, Tokyo, Japan) was mounted through the top aperture of the acquisition chamber at *ca.* 55 cm from the roots. All image enhancement settings were set to ‘off’ or ‘neutral’. The inside was coated with a reflective surface and contained six light emitting diode (LED) panels with a colour temperature of 5500° K (Illuminant D55, 93+ colour rendering index, close to white mid-morning/mid-afternoon daylight). The imaging chamber provided constant light conditions and uniform distance from the lens and camera focus. Two light environments were created: 1) *without filter* (WF), consisting of four LED panels (two per side) at 30–40° and 60–70° angles, respectively; and, 2) *polarisation filter* (PF), consisting of one LED panel per side covered with 3 mm acrylic glass (including a 5 mm ventilation gap) at a 30–40° angle, carrying a linear, 0.3 mm polarisation foil (B+W Filter, Schneider, Bad Kreuznach, Germany). In this setup, a polarisation filter (XS-Pro Digital HTC Polfilter KSM MRC nano, 77 mm, B+W Filter, Bad Kreuznach, Germany) was also mounted on the lens. The polarisation setup reduced the reflections produced by moisture on the surface of the samples.

The resulting light intensity at the sample surface was not directly measured; instead, relative exposure settings were systematically varied under controlled conditions to assess their effect on colour–pigment relationships. The two light environments, *WF* and *PF*, were combined with three relative exposure steps (of -1, 0, + 1, and +2) corresponding to different exposure times, creating six different light conditions: *WF –1* (1/25 s), *WF 0* (1/50 s), *WF +1* (1/100 s), *PF 0* (1/100 s), *PF +1* (1/60 s), and *PF +2* (1/30 s). Throughout this report, the *WF 0* is selected, and subsequently referred to, as the *default light condition* because it best resembles the basic starting set-up of digital photography and accessible artificial lighting. It is thus used in pattern recognition of the data (pre- and post-statistical analysis), which will be referred to in following sections *3 Results* and *4 Discussion*.

Immediately after harvest, six carrots per cultivar (96 total) were cut in half crosswise and a disk (4 mm thick) was sliced from the central region of the root to standardise sampling and reduce variability associated with positional differences along the root. All six disks of the same cultivar were placed in the image acquisition chamber at the same time. The carrot disks were placed on a blue background and photographed under each light condition using a remote-control clicker to capture the images. A colour checker (ColorChecker Passport Photo 2, X-Rite, Grand Rapids, MI, USA) was present in each image for colour calibration (**Figure 1A**). The remaining portions of each root were labelled and stored in dark plastic bags at 4 °C to mimic underground conditions and avoid the degradation of the pigments until pigment extraction (Cinar, 2004).

### 2.3 Image analysis: CIELab/Ch colour parameters

All images were analysed using FIJI (ImageJ) version 2.14.0/1.54f (Schindelin et al., 2012). The colour checker (ColorChecker Passport Photo 2, X-Rite, Grand Rapids, MI, USA) was used to calibrate the images with the IJP-Colour Calibrator plugin. For replicability: the plugin-specific specifications were set as: X-Rite passport with 20% chip margin and magenta chip overlay colour (overlay stroke width of 1 unit), all chips enabled. The combination of a standard red, green, and blue (sRGB; standardised colour space for consistent colour across devices) reference and quadratic cross-band mapping method resulted in the most informative results and the smallest mean delta for calibration (higher colour accuracy).

The perimeter of each carrot disk was highlighted once by hand using the polygon selector tool, and the same region of interest was then automatically applied to the corresponding samples under the other five light conditions (i.e., a FIJI macro was created to automate the analysis per sample). It was the most precise way to highlight the flesh as close to the root edge as possible while avoiding the unwanted peel and background (see: **Supplementary Figure 1**). A colour histogram was performed per sample and the mean ± standard deviation of red, green, and blue (RGB) values recorded.

The RGB colour values obtained were converted into the CIELab/Ch colour space for device-independence and compatibility with current literature (e.g., Goñi and Salvadori, 2017; Agarwal et al., 2021). The mathematical transformations were based on the *rgb2lab* function from Skimage (scikit-image) open-source Python library (Van der Walt et al., 2014). The intensities of the red, green, and blue channels were converted into L*, a*, and b* colour parameters via CIE *XYZ* (Phillips, 2023). Despite using a D55 illuminant in image acquisition, calculations were performed for the D65/10° standard illuminant (see: Phan Van Song, 2018). The parameters C* and *h°* were calculated using the following Equations 1, 2:

(1) $$C^{*}={\left[{\left(a^{*}\right)}^{2}+{\left(b^{*}\right)}^{2}\right]}^{1/2}$$

(2) $$h{}^{\circ}=arctan\left(b^{*},a^{*}\right)$$

### 2.4 Pigments extraction and quantification

#### 2.4.1 Total carotenoids

Total carotenoid content (TCC) quantification was performed following the method by Moreno and Stange (2022). The remaining portions of the same roots used for image acquisition (*n* = 6 per carrot cultivar) were individually ground, snap-frozen in liquid nitrogen and stored at -80 °C immediately after image acquisition. Then, the samples were freeze-dried at -55 °C using the benchtop freeze dryer (Alpha 1–4 LSCplus, Martin Christ, Osterode, Germany) until weight was stable. Samples were homogenised with the bead ruptor (OMNI Bead Ruptor 24 Elite, OMNI International, Kennesaw, GA, USA) and stored at -80 °C to prevent degradation of the pigments. For extraction, homogenates were centrifuged at 2200 x g for 5 min at 4 °C in 1 mL 100% pure acetone three times until pellets were colourless. Spectrophotometric determination of TCC in the supernatants was performed with the microplate reader (Synergy HTX, BioTek Instruments, Winooski, VT, USA) at 25 °C in a quartz 96-well microplate (Hellma Analytics, Müllheim, Germany) within the spectrum of visible light (390–710 nm) according to the Lichtenhaler equations (Lichtenthaler and Wellburn, 1983).

The absorbance of the solution at wavelengths of 662, 645, and 470 nm was used to calculate the chlorophyll (*C_a_ + C_b_*, Equations 3, 4*)* content, from which TCC (*C_x+c_*, Equation 5) can be obtained. The latter is indicated in µg/mL.

(3) $$Chlorophyll\;a:C_{a}=11.75A_{662}-2.35A_{645}\left(\mu g/ml\;solution\right)$$

(4) $$Chlorophyll\;b:C_{b}=18.61A_{645}-3.96A_{662}\left(\mu g/ml\;solution\right)$$

(5) $$Total\;carotenoids:C_{x+c}=\left(1000A_{470}-2.27C_{a}-81.4C_{b}\right)/227$$

#### 2.4.2 Individual carotenoids

Freeze-dried samples (n = 5 per carrot cultivar) were ground and extracted with a heptane:methanol:acetone solvent (3:1:1, v/v/v) containing 1 g/L butylated hydroxytoluene (BHT). Samples were placed in an ultrasonic bath (10 minutes), then shaken at 2500 rpm (20 minutes), and centrifuged at 4600 rpm (5 minutes) followed by removal of the upper heptane layer. The extraction was repeated. The combined heptane layers were mixed with ethanol. Samples were filtered through a 0.22 µm syringe filter.

Chromatographic separation was performed on a YMC C30 column (250 mm × 4.6 mm, 3 µm particle size) maintained at 20 °C. The mobile phase consisted of three eluents: (A) methanol (98:2, v/v), (B) methanol (95:5, v/v), and (C) tert-methyl butyl ether. A gradient elution was employed at a flow rate of 1.0–1.4 mL/min. Step one (80% A and 20% C) was stopped after 2:00 min; step two (decreased A to 60% and increased C to 40%) lasted one second; and step three (60% B, 40% C) was stopped at 12:00 min. The column was washed with 100% C for from timepoints 12:00 to 13:00 min (steps four and five), before re-equilibration for steps six and seven (80% A and 20% C) for 6:59 seconds. The total run time was 20:00 minutes.

A photodiode array detector was used with a wavelength range of 300–600 nm and 2 nm spectral resolution. Carotenoid detection was fixed on a 450–470 nm range with a 4 nm resolution. Calibration curves for lutein, lycopene, and β-carotene were calculated with authentic carotenoid standards. α-carotene was measured using the relative response factor of β-carotene.

#### 2.4.3 Total monomeric anthocyanin

The plant material that remained of the red and purple carrot cultivars was thawed and immediately used for monomeric total anthocyanin content (TAC) quantification (six samples for cultivars P1 and PY1, three samples for cultivar P2, and one sample for cultivar R1). The pH differential method was used to determine TAC (Pérez et al., 2022; Baldassi et al., 2024). The protocol followed is accessible in: https://doi.org/10.34894/BTPTSV. Extraction was performed in duplicate by grinding the samples with 70% ethanol containing 1% HCl (v/v) three times until the pellet was colourless. Half of the supernatant was diluted in a 25mM KCl solution (pH: 1), and the other half in a 400mM sodium-acetate solution (pH: 4.5), both at a 1:40 dilution factor.

Both solutions were analysed with the same spectrophotometric equipment and wavelength range (390–710 nm) as described for TCC quantification. Distilled water was used as the blank. The absorbance at 520 and 700 nm for both solutions was used to calculate TAC per sample with Equations 6, 7, where *A* = absorbance, *MW* = cyanidin 3-glucoside molar weight, *DF* = dilution factor, *EF* = cyanidin 3-glucoside extinction factor, and 1000 is the conversion factor from g to mg. Results were then converted into mg/100 g FW using the average moisture content per cultivar.

(6) $$Absorbance:A=\left(A_{520}-A_{700}\right)pH:1.0-\left(A_{520}-A_{700}\right)pH:4.5$$

(7) $$Total\;anthocyanins:TAC=\left(A*MW*DF*1000\right)/EF\left(mg/L\right)$$

### 2.5 Statistical analysis

Statistical analyses were performed using R software (R core team, 2021), version 4.3.2. Single and multiple linear regressions were modelled using the L*, a*, b*, C*, and *h°*colour parameters as predictors of TCC, TAC, lycopene, lutein, α-carotene, and β-carotene individually. Multiple linear regressions were performed with all the colour parameters to observe the effect of the five colour predictors combined (and for reproducibility across the board).

Ultimately, partial least squares (PLS) regressions were modelled from the CIELab/Ch colour parameters for prediction of TCC, TAC, lycopene, lutein, α-carotene, and β-carotene. The observations were standardised by subtracting the mean value of each variable and dividing that by its standard deviation, creating variables with a mean of zero and variance of one (z-score normalisation). Five components (latent variables) were created from the predictors to maximise the covariance between the colour parameters and the pigment content and handle the high multicollinearity between predictors. Standardising the observations ensures that colour variables with large variance do not dominate the first (heaviest) components, thus masking variables with lower variances. Data was split into ten random segments, alternately using nine for calibration and one for validation (prediction). The pigment content predictions were then reverted to their original scales and compared to the actual pigment levels to evaluate the average predictive power of the models using the root mean square error of prediction (RMSEP) (see also: **Supplementary Figure 3**).

## 3 Results

### 3.1 Colorimetric image analysis

**Figure 2:**
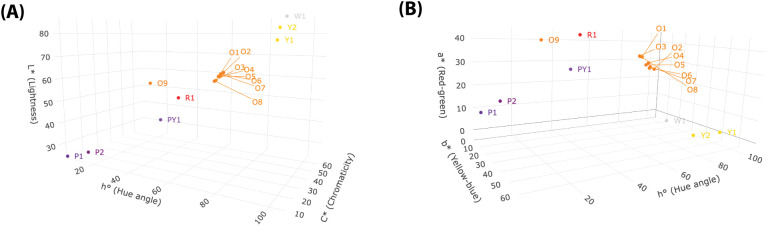
Location of the different carrot cultivars on the CIELab/Ch colour space according to their mean root flesh colour represented by the three colour parameters **(A)** L* (Lightness), C* (Chromaticity) and *h°* (hue angle) as axes and **(B)** a* (redness), b* (yellowness) and *h°* (hue angle) as axes. The cultivars classified in the CIELab/Ch colour space correspond with four main colour groups identified as: white, yellow, purple, and orange/red.

The location of the carrot cultivars, based on each cultivar’s average colour values, is described three-dimensionally with CIELab/Ch parameters L*, C* and *h°* (**Figure 2A**) and a*, b* and *h°* (**Figure 2B**). The cultivars classify into four main clusters of colours (purple, orange/red, yellow, white), despite changing the colour space/parameter composition. The colour of the fully purple cultivars (P1, P2) comprise low values for all parameters; the white (W1) has low a* and b* values, but high L* and *h°*; the yellow cultivars (Y1, Y2) have low a* and high b*, and, on the contrary, the orange/red group has high a* and low b*. The purple carrots with yellow cores (cultivar PY1) group amongst the orange/red cultivars.

### 3.2 Total pigment content quantification

**Figure 3:**
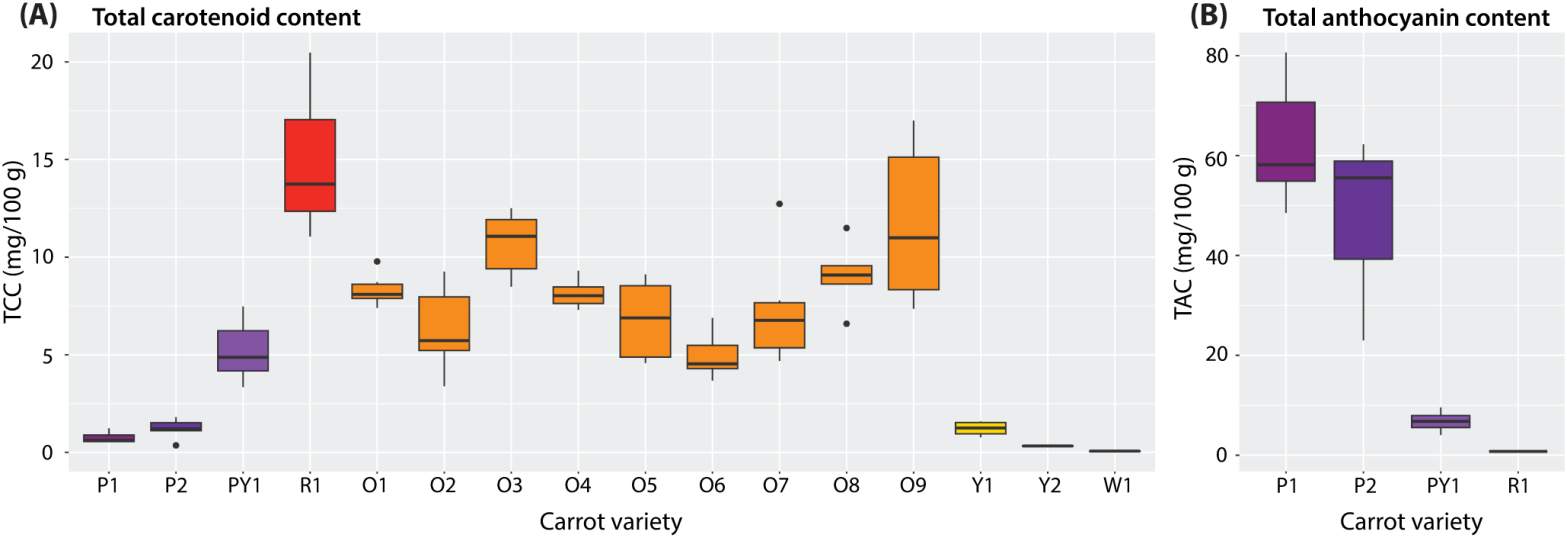
**(A)** Total carotenoid content (mg/100 g) per carrot cultivar in fresh weight (FW); **(B)** total anthocyanin content (mg/100 g, FW) per carrot cultivar. Purple cultivars (P1, P2); purple-yellow cultivar (PY1); red cultivar (R1); orange cultivars (O1–O9); yellow cultivars (Y1, Y2); and white cultivar (W1).

Regardless of their colour and cultivar, the carrots exhibited significant variability in carotenoid content (FW), spanning from 0.04 (W1) to 20.48 (R1) mg/100 g (**Figure 3A**). The red cultivar (R1) had the highest average TCC, at 14.83 (± 3.65) mg/100 g. The greatest variability of TCC *within* each cultivar was found amongst the orange roots (O1-O9), of which O9 carrots differed as much as 8.97 mg/100 g, followed by O2 which differed as much as 5.88 mg/100 g within the cultivar. The most stable cultivars amongst the orange roots were O1, O4, and O8 with average values *ca.* 8.5 mg/100 g. White, yellow, and fully purple carrots contained minimal TCC levels, with the exception of the purple/yellow cultivar (PY1), which had on average 5.20 (± 1.57) mg/100 g - comparable to levels of the orange cultivars.

TAC amongst purple and red carrots varied from 0.77 mg/100 g in the red cultivar (R1) and 80.65 mg/100 g in a purple cultivar (P1) (**Figure 3B**). High TAC variability can be observed amongst the fully purple roots (P1, P2) and low content-low variability in the PY1 cultivar. The red carrot was excluded from further TAC analyses due to low content and insufficient samples.

### 3.3 Modelling total pigment content with CIELab/Ch colour parameters under different experimental light conditions

#### 3.3.1 Preliminary single and multiple linear regressions

**Figure 4:**
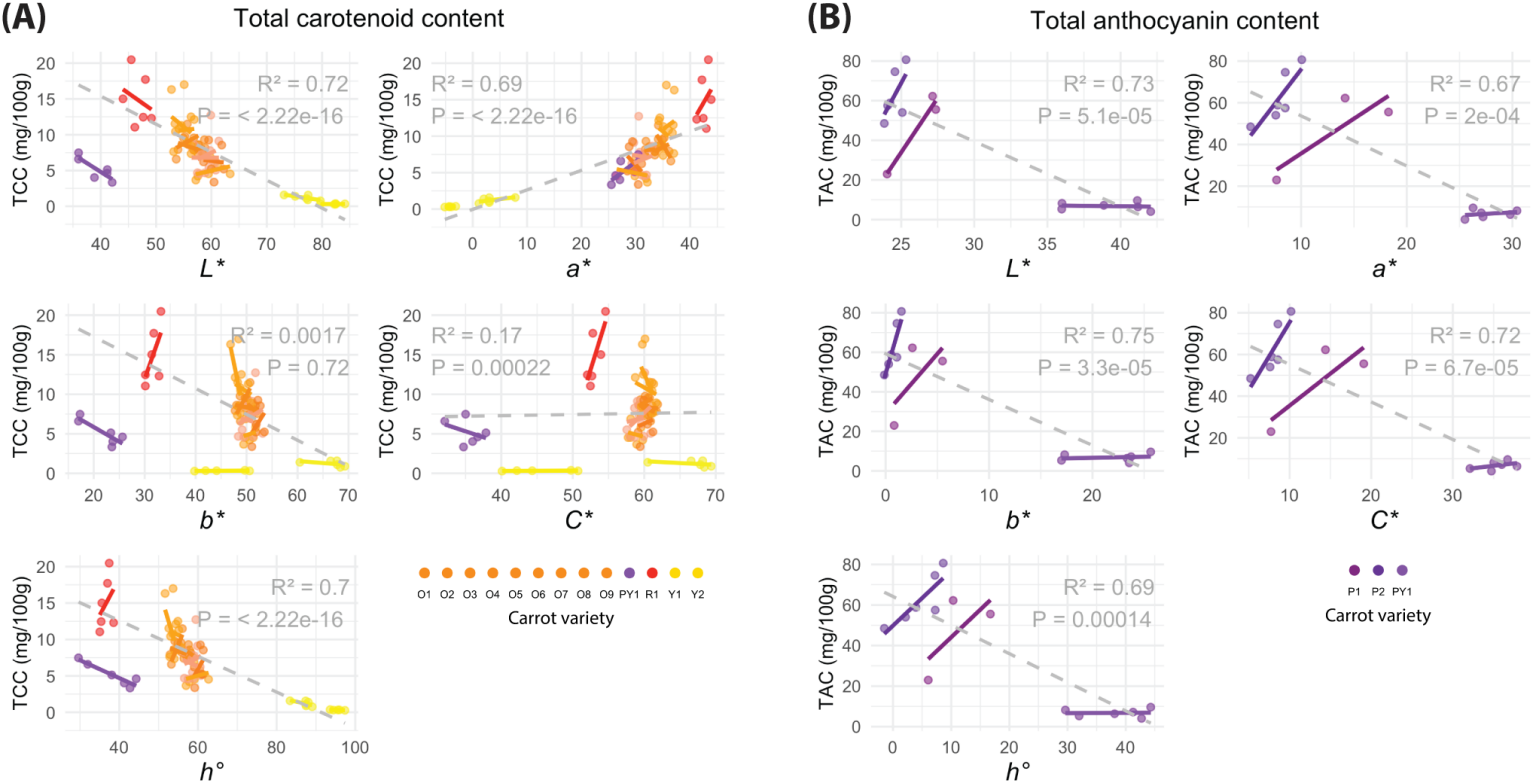
Single linear regressions performed between each individual CIELab/Ch colour parameters (L*, a*, b*, C* and *h°*) and **(A)** total carotenoid content (mg/100 g) and **(B)** total anthocyanin content (mg/100 g). Colour analysed from images taken under the default, without filter (*WF 0*), light condition.

**Figures 4A, B** shows the relationship trends between the individual CIELab/Ch colour parameters and TCC, as well as TAC, under the default *WF 0* light condition. The multiple linear regression results for the total pigment contents and all the CIELab/Ch colour parameters under the six light conditions are shown in **Table 1**. The coefficients of determination (*R^2^*) of the regressions range between 0.72–0.78 (TCC) and 0.68–0.76 (TAC) (*P-value* < 0.01). One can observe how the individual [significant] contributions of each colour parameter on total pigments prediction varied greatly depending on the light condition used during image acquisition, especially for TCC (**Table 1**).

**Table 1:** Correlation matrix of multiple linear regressions performed from all CIELab/Ch colour parameters (L*, a*, b*, C* and *h°* together) as predictors of total carotenoid content and total anthocyanin content individually, classified by experimental light condition (*WF =* without filter, *PF* = polarisation filter) ranging from -1 to +2 in exposure steps.

Total carotenoid content						
Parameters	*WF -1*	*WF 0*	*WF +1*	*PF 0*	*PF +1*	*PF +2*
L*	0.141	0.109	-0.102	-0.26	-0.969***	-0.507**
a*	0.363	0.321	0.085	0.1	-0.165*	-0.058
b*	-0.519**	-1.089**	-0.818*	-0.789**	-0.003	-0.111
C*	0.595**	1.23**	0.926*	0.872*	0.011	0.097
*h°*	-0.188	0.342*	0.155	0.248	0.164	0.002
**R²**	**0.769**	**0.716**	**0.756**	**0.769**	**0.751**	**0.778**
P-value	< 0.001	< 0.001	< 0.001	< 0.001	< 0.001	< 0.001

Total anthocyanin content						
Parameters	*WF -1*	*WF 0*	*WF +1*	*PF 0*	*PF +1*	*PF +2*
L*	1.397	-0.221	0.725	2.618	-4.8	-5.194
a*	-16.924	-32.545	3.348	-42.987	1.653	-0.866
b*	-15.34	-25.483	5.937	-37.433	2.351	-1.327
C*	21.309	41.031	-7.784	55.054	-2.336	2.407**
*h°*	0.344	0.325	-1.995	0.665	-0.185*	-0.258
**R²**	**0.671**	**0.748**	**0.753**	**0.763**	**0.726**	**0.674**
P-value	< 0.001	< 0.001	< 0.001	< 0.001	< 0.001	< 0.001

Explained variance (*R^2^*); *P-value* < 0.001 (***); *P-value* < 0.01 (**); *P-value* < 0.05 (*).Bold values represent the coefficient of determination (R^2^) of the model.

#### 3.3.2 Partial least squares regressions

Results of a partial least squares (PLS) regression (**Table 2**) exhibit consistently strong relationships of all CIELab/Ch colour parameters with TCC and TAC (*R^2^* = 0.734–0.793 and *R^2^* = 0.781–0.842, respectively) across all light conditions. Despite producing high *R^2^* values, models with relative root mean square errors of prediction (RMSEP) values above 55% are considered inoperable. The model obtained from the *PF +2* light condition had the highest predictive power for TCC with a strong explained variance (*R^2^* = 0.793) and the lowest RMSEP of 2.214 mg/100 g (31.7%). However, TAC prediction was unfavourably influenced by this light condition, resulting in an extremely high RMSEP of 145.2%. Therefore, *WF 0* and *PF 0* are identified as the most useful light conditions for the prediction of both total pigment contents (see **Table 2**).

**Table 2:** Correlation matrix of partial least squares regressions for the prediction of total carotenoid content and total anthocyanin content using the CIELab/Ch colour space, classified by experimental light condition (*WF =* without filter, *PF* = polarisation filter) ranging from -1 to +2 in exposure steps.

Light condition	R²	Calibration		Cross-validation	
		RMSE	(%)	RMSEP	(%)
Total carotenoid content					
*WF -1*	0.734	2.388	34.2	5.525	79
*WF 0*	0.784	2.153	30.1	2.266	32.4
*WF +1*	0.772	2.211	31.6	2.352	33.6
*PF 0*	0.784	2.153	30.1	2.303	32.9
*PF +1*	0.768	2.233	31.9	6.937	99.2
*PF +2*	0.793	2.108	30.2	2.214	31.7
Total anthocyanin content					
*WF -1*	0.781	13.11	35.4	22.14	59.8
*WF 0*	0.824	11.51	30.1	15.09	40.7
*WF +1*	0.835	11.37	30.7	18.8	50.8
*PF 0*	0.842	11.13	30.1	14.9	40.2
*PF +1*	0.818	11.96	32.3	70.47	190.3
*PF +2*	0.783	13.03	35.2	53.75	145.2

All *R^2^* values are statistically significant (*P-value* < 0.001). Explained variance (*R^2^*); root mean square error (RMSE) used during training of model (calibration); root mean square error of prediction (RMSEP) used during cross-validation.

(*WF 0*) PLS equations of the relationship between the CIELab/Ch colour space for TAC (Equation 8) and TCC (Equation 9):

(8) TAC=0.524L*−255.3a*−239.0b*+437.4C*+25.2h

(9) TCC=−1.71L*+2.56a*−9.70b*+7.90C*+5.43h°

(PF 0) PLS equations of the relationship between the CIELab/Ch colour space for TAC (Equation 10) and TCC (Equation 11):

(10) TAC=20.1L*−458.5a*−402.6b*+794.5C*+10.0h°

(11) TCC=−2.77L*+2.65a*−11.2b*+9.31C*+6.55h°

### 3.4 Individual carotenoid content quantification

**Figure 5:**
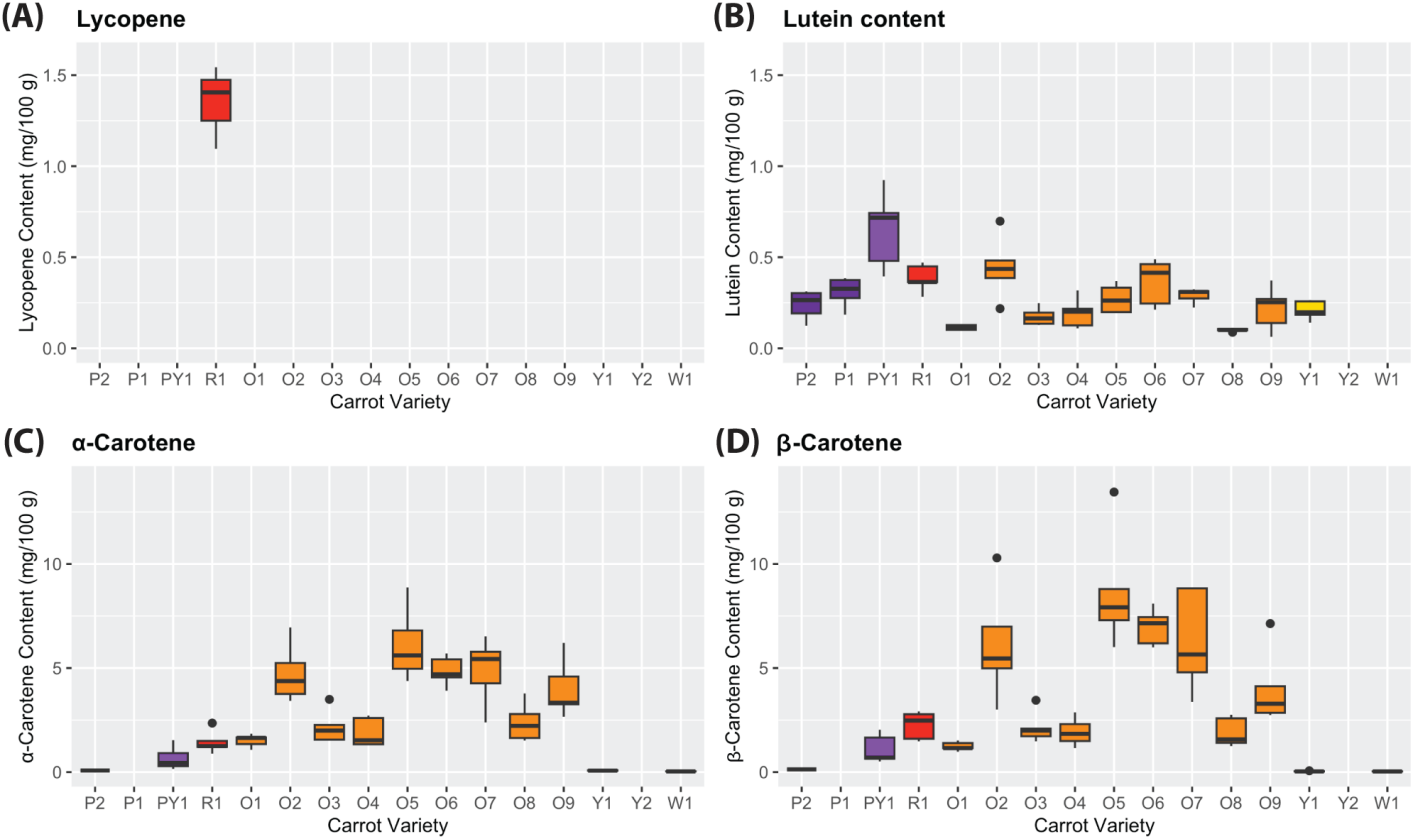
Content of **(A)** lycopene, **(B)** lutein, **(C)** α-carotene, and **(D)** β-carotene (mg/100 g) per cultivar in fresh weight (FW). **(A)** and **(B)** have a scale of 0.0–1.7 (mg/100 g) and **(C)** and **(D)** have a scale of 0–15 (mg/100 g). Purple cultivars (P1, P2); purple-yellow cultivar (PY1); red cultivar (R1); orange cultivars (O1–O9); yellow cultivars (Y1, Y2); and white cultivar (W1).

Individual carotenoids content was analysed by HPLC per carrot cultivar (n = 5) (**Figures 5A–D**). Lycopene was found only in the red cultivar (R1) (average of 1.66 ± 0.45 mg/100 g). Lutein content was particularly stable among all cultivars. The purple/yellow cultivar (PY1) contained the highest lutein content (average 0.65 ± 0.21 mg/100 g), while yellow (Y2) and white (W1) cultivars contained levels below 0.025 mg/100 g. As was expected, all orange cultivars were found to contain the highest values of α-carotene and β-carotene. Some orange cultivars (O2, O5, O6, O7, and O9) contained the most elevated β-carotene content and also the most variability of it *within* each cultivar. The β-carotene content of the purple/yellow cultivar (PY1) more closely resembles the orange cultivars than the other purple ones. Overall, the white (W1), yellow (Y2, Y1), and fully purple (P1, P2) cultivars contained negligible lycopene, α-carotene, and β-carotene contents.

### 3.5 Modelling individual carotenoids content with CIELab/Ch colour parameters

**Figure 6:**
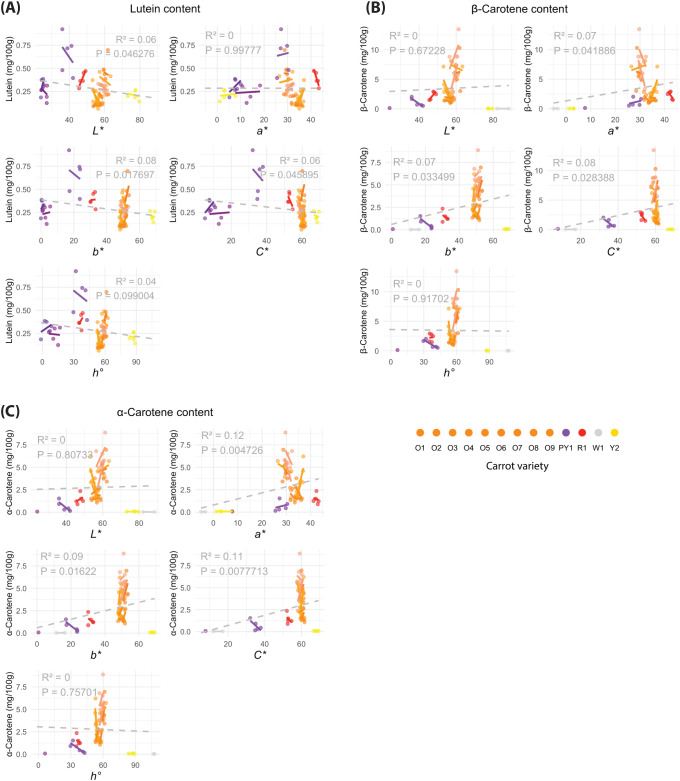
Single linear regressions between the individual CIELab/Ch colour parameters (L*, a*, b*, C* and *h°*) and **(A)** lutein; **(B)** α-carotene; and **(C)** β-carotene contents (mg/100 g) per cultivar. Colour analysis performed on images taken under the default, without filter (*WF 0)*, light condition.

Given the high-precision HPLC quantification, there was no there was no minimum pigment threshold, only considered in the limit of the device detection (0.025 mg/100 g), to include samples into the statistical analyses. Only the fully purple cultivars (P1, P2) were omitted to reduce the noise that occurs from samples with strong colour values but relatively minimal individual carotenoid content. No relationship was found between colour and lycopene because only the red cultivar (R1) was found to contain the pigment (**Figure 5A**). All CIELab/Ch colour parameters resulted in poor, though significant (*P-value* < 0.01), single linear relationships with lutein, α-carotene, and β-carotene content (except for lutein–a* and β-carotene–b* which were insignificant) (**Figures 6A-C**).

As described in *section 2.2*, the *WF 0* light condition is considered the default environment and was used in the creation of PLS regressions between the individual carotenoid contents and colour (all CIELab/Ch colour parameters). The different models resulted in *R^2^* values between 0.428 (for β-carotene) and 0.488 (for lutein) (**Table 3**). The lowest relative RMSEP (rRMSEP; RMSEP relative to the measured values) was obtained from the model for lutein (34.1%), followed by α-carotene (52.4%) and β-carotene (60.1%). Given the low relationships, PLS models for α-carotene and β-carotene estimation were tested by including only the orange cultivars. Since these resulted in similarly-fit models (*R^2^* = 0.428, rRMSEP = 49.9%; and *R^2^* = 0.470, rRMSEP = 60.9%, respectively), there is no reason to deviate into a smaller sample group and explain them further.

**Table 3:** Partial least squares (PLS) models for the prediction of different pigment contents using the CIELab/Ch colour space to analyse images taken under the default, without filter (*WF 0*), light condition.

Pigment	Content range (mg/100 g) FW	PLS equation	R²	RMSEP (cross-validation)	
				Estimation of error (mg/100 g)	(%)
Total anthocyanins	4.03 – 80.5	= 0.524L* – 255.3a* – 239.0b* + 437.4C* + 25.2*h°*	0.824	15.09	40.7
Total carotenoids	0.28 – 20.5	= 1.211L* + 4.877a* – 13.41b* + 16.13C* + 6.480*h°*	0.784	2.27	32.4
Lutein	0.25 – 0.92	= -0.01415L* – 0.06352a* – 0.04511b* – 0.07839C* – 0.003790*h°*	0.488	0.13	34.1
α-carotene	0.25 – 8.88	= 0.02682L* – 2.898a* – 12.42b* + 9.467C* + 4.052*h°*	0.435	1.63	52.4
β-carotene	0.25 – 13.46	= 0.354L* – 5.917a* – 24.53b* + 19.89C* + 6.291*h°*	0.428	2.39	60.1
Lycopene	0.25 – 2.06	*Not enough presence to model*			

Root mean square error of prediction (RMSEP) used to cross-validate the model; fresh weight (FW); explained variance (*R^2^*).

## 4 Discussion

Literature on pigment prediction via colorimetric assessment frequently accepts simple correlations between carotenoids/anthocyanins and individual colour parameters (Meléndez-Martínez et al., 2003; Montes et al., 2005; Moyano et al., 2008; Eoh et al., 2025). However, individual colour parameters contribute to multi-dimensional colour spaces, such as CIELab/Ch, and thus are highly multicollinear variables. This study tested the replicability of such approaches and attempted to develop a more robust image analysis optimisation (IAO) for the assessment of pigment content in carrots with roots of high genotypic variety. To the best of our knowledge, the relationship between CIELab/Ch colour parameters and total carotenoid content (TCC) and/or total anthocyanin content (TAC) has not been investigated in this manner before, nor with a group of carrot cultivars ranging from white to purple in colour. Literature is especially poor regarding relationships between colour and TAC.

### 4.1 The importance of image acquisition and pre-processing

In this IAO, the colour of samples was analysed from digital images by obtaining the average colour of each carrot disc, taken from the middle of the root, and used to represent the entire carrot. Wet lab quantifications using spectrophotometric and HPLC analyses also considered the average pigment content per carrot since they were performed from homogenates of the same samples. Although the uniformity of both flesh colour and pigment distribution may vary, Reeves (1987) observed higher colour-pigment correlations from the colour of pureed paprika samples than the fruit whole, as was considered in this study both in average colour and homogenate wet lab analyses. It is additionally advantageous to analyse the average colour of a carrot disk in the interest of maintaining the IAO a non-destructive method by nature.

Image-based colorimetric approaches often assume that controlling lighting conditions is sufficient, but do not explicitly account for how illumination and camera response influence measured colour values, which can affect reproducibility (Agarwal et al., 2021; Kim and Van Iersel, 2023). However, our results showed that the aperture and exposure time of the digital camera changed despite maintaining the light environment the same (see: **Supplementary Figure 2**), thus requiring calibration per light condition (i.e., *WF 0*, *WF +1*, *PF 0*, etc.). Colour differences between the carrot cultivars, a product of the genotypic diversity in this study, have a significant influence on the light registered by automatic digital cameras, even when specific settings are specified. This occurs because darker roots require a longer exposing time where lighter carrots require less light. Colour calibration therefore determines the validity and replicability, regardless of the camera brand/model and image acquisition conditions (e.g., automatic aperture, photography chambers, and other standardised aids). Meléndez-Martínez et al. (2003) found that levels of different carotenoids were better correlated to individual CIELab/Ch parameters when measured (with a tristimulus colorimeter) from samples laid on a black background compared to a white one. Given the strong influence of environmental light (colour and intensity), the effect of six different light conditions (unpolarised *WF -1, WF 0, WF + 1*, and polarised *PF 0, PF + 1*, and *PF +2*) on the relationship between colour and pigment content was assessed in this study. Throughout this report, *WF 0* is considered the default light condition because it most similarly resembles a standard, accessible set-up. The successful results of this lighting condition are discussed later.

### 4.2 Carrot colour and pigment content variability

The CIELab/Ch values of the samples were found to correspond with their visual colour. This allows categorisation of the different cultivars into four distinct groups: white, yellow, purple, and orange-red (**Figure 2**). The latter includes the PY1 cultivar (i.e., purple with yellow core), which has a TCC level closer to that of the orange/red cultivars than to the other purple ones. Thus, it is interpretable that this IAO was able to capture colour-pigment intricacies found in the flesh of the PY1 roots to some degree.

The content levels of total and individual carotenoids quantified in this study (**Figures 3A**, **5**) are in line with literature (Surles et al., 2004; Jourdan et al., 2015). The sum of the lycopene, lutein, α-carotene, and β-carotene contents is equal to the TCC of each sample, thus validating the latter’s spectrophotometric quantification. The TAC of all purple cultivars (**Figure 3B**) is also within the standard range for dark carrots (Kammerer et al., 2004; Cuevas Montilla et al., 2011). Samples containing less than 0.25 mg/100 g of total carotenoid and total anthocyanin content were omitted from further analyses for each pigment group. This is because false positives are conceivable from strongly-coloured cultivars that in reality contain minimal levels of a given pigment (i.e., strong-coloured purple cultivars with minimal TCC). Regarding individual carotenoids content, however, non-purple carrot cultivars containing less than 0.25 mg/100 g *were* included because they were quantified with high precision via HPLC and did not alter the results significantly. Lycopene was omitted from further analyses after identifying its minimal presence amongst the carrot samples (**Figure 5A**), as was done by Jourdan et al. (2015) in their study of yellow-to-orange carrots.

### 4.3 Traditional colour-pigment predictions: limitations of single/multiple linear regressions

An adequately strong negative relationship between the CIELab/Ch parameter of colour L* and both TCC (*R^2^* = 0.72) and TAC (*R^2^* = 0.74) is observed in **Figures 4A, B**, as traditionally reported from studies on different fruits/vegetables (Singha et al., 1991; Meléndez-Martínez et al., 2003; Han et al., 2008; Itle and Kabelka, 2009; Dóka et al., 2013).

Evidently, dark-coloured samples (lower L* value) contain higher pigment content than samples with little to no colour at all (e.g., white roots). However, L* is the only colour independent (self-standing) colour parameter and, therefore, does not provide enough information to robustly estimate pigment content within a population of high genotypic variability. For instance, two carrot samples, one from the purple/yellow cultivar (PY1) and one from the red (R1), have similar L* values (42.07 and 44.01, respectively), yet the PY1 contains only 22.2% of the TCC found in the R1 (**Figure 4A**). Additionally, single linear regression results in the literature are undisputedly contradictory, despite exhibiting very favourable results (*R^2^* = 0.903– 0.999) (Meléndez-Martínez et al., 2003; Hyman et al., 2004; Seroczynska et al., 2006; Dóka et al., 2014; Vieira et al., 2019; Eoh et al., 2025).

The other CIELab/Ch colour parameters (a*, b*, C* and *h*) exhibit high degrees of multicollinearity considering that a* and b* are orthogonal coordinates (representing the green-red and blue-yellow axes) and C* and h° are mathematically derived from them. Since 1983, Munsch, Simard and Girard suggested to consider all parameters of multi-dimensional colour spaces to capture sample colour accurately and thus be able to correctly estimate pigment content. However, 2004–present studies still disregard this multi-dimensional property (i.e., use single linear regressions) or only partially consider it (i.e., multiple linear regressions).

Multiple linear regressions are not fully compatible with CIELab/Ch colour parameters because of the emergent ‘zone of confusion’ suggested by Eagerman et al. (1973). It is an area within each colour parameter scale where, after a certain threshold of pigment accumulation, colour changes less dramatically, which suggests a non-linear relationship (Eagerman et al., 1973). Both Ruiz et al. (2008) and Seroczynska et al. (2006) combined several CIELab/Ch colour parameters and reported stronger correlations with TCC in samples with lower pigment concentrations than those highly pigmented. A possible explanation, described by Stahl and Sies (2003) is that the chemical structure of carotenoids (e.g., number of covalent double-bonds) influences the intensity of colour they emit, which varies between the different carotenoids (e.g., β-carotene has more covalent double-bonds and a higher effect on coloration than most other carotenoids). Similarly, some anthocyanins emit more perceivable colour than others, such as cyanidin-3-glucoside, producing great changes in colour values from smaller variations in concentration (Han et al., 2008). Therefore, after a certain threshold of pigment content, it is hard to discern between the impact/influence of the pigment on a given sample’s colour (Eagerman et al., 1973).

In the current study, traditional single and multiple linear regressions were used to demonstrate that single colour parameters are not enough to accurately estimate total pigment content (**Figures 4**, **6**). Performing multiple linear regressions with all CIELab/Ch colour parameters showed that the weight of each parameter in the model varies depending on the light condition used during image acquisition (**Table 1**). This suggests that the results obtained in previous studies might be highly influenced by their unreported light conditions/environment.

### 4.4 Addressing multicollinearity in colour-pigment relationships: partial least squares (PLS) regression

The advantages of PLS regressions are the standardisation and creation of latent variables to correct for multicollinearity between predictors, as suggested by some researchers in the field (Meléndez-Martínez et al., 2003; Ruiz et al., 2008; Moresco et al., 2017). The use of PLS generated very satisfactory correlations between carrot root colour and TCC (*R^2^* = 0.73–0.79) as well as TAC (*R^2^* = 0.73–0.84) despite the light condition (**Table 2**). The PLS models were calibrated and cross-validated using all the data by splitting it into ten random segments and alternately using nine for calibration and one for validation. This enables the assessment of model accuracy with ‘new’ data. Though the PLS results in this report exhibit lower explained variance values than other studies using single and multiple linear regressions, the outcome reveals that performing robust statistics on colour analyses may result in stable estimation of pigment contents in variable light environments. Consequently, this approach to pigment content prediction may be explored outside of standardised laboratory conditions (e.g., in the field) and be better extrapolatable and practical.

#### 4.4.1 Effect of light conditions on the model

The best-fit models for both TCC and TAC are those performed under *WF 0* and *PF 0* light conditions, as they have satisfactory *R^2^* values and the lowest root mean square error of prediction (**Table 2**). The four models slightly underestimate the actual pigment content (**Supplementary Figure 3**). Although rRMSEP values of 30.2–30.4% (TCC) and 40.2–40.7% (TAC) account for seemingly high error on new data, the values are a product of the genetic variability found within the different carrot cultivars as can be observed in **Figures 3**, **4**. Similar relative prediction error values have also been reported by others (Merzlyak et al., 2003; Ruiz et al., 2008; Moresco et al., 2017). Indeed, the risk/consequence of such investigations is not particularly high, and the pigment content is measured in mg/100 g, suggesting that the range of possible prediction (error) is actually narrow (**Table 3**). Additionally, image analyses do not have to be stand-alone assessments of pigment content or food quality but may assist or facilitate other procedures. Thus, their development is encouraged. For instance, Rodríguez-Pulido et al. (2017) combined digital images of grape samples with their spectral image equivalents to successfully assess sample quality better than with the two methods independently. Although our methodology is not capable of predicting individual carotenoids (*further explanation below*), it very satisfactorily identifies TCC and TAC in a way that, if combined with other methodologies, could facilitate and decrease the costs of traditional laboratory analyses.

#### 4.4.2 The multi-dimensional image analysis model

With the intention of identifying the best model for TCC and TAC estimation obtained in this study, the *WF 0* light condition was selected (rather than the similarly-fitting *PF 0*) given that it most closely resembles a default camera and lighting set-up: a more accessible and replicable model. Different combinations of the CIELab/Ch colour parameters (e.g., (a*/b*) and (a*/b*)^2^ ratios, etc.) were tested under the given light condition as suggested by literature, however the full colour parameter profile rendered the best fit for all pigment groups in this study (Hyman et al., 2004; Seroczynska et al., 2006; Moyano et al., 2008; Kljak et al., 2014). This clearly demonstrates the multi-dimensionality of the pigment-colour relationships.

**Table 3** shows the PLS models for the different pigments under the selected *WF 0* light condition. Although the PLS models for individual carotenoids prediction (lycopene, lutein, α-carotene, and β-carotene) did not obtain satisfactory results (*R^2^* = 0.428–0.488), **Figures 5**, **6** reveal that there exists a high variability of phenotypic expression of the pigments within the carrot cultivars themselves. In this study, that is not a consequence of environmental variability given that the 96 samples were grown and sowed under the same conditions and on the same dates. Therefore, genetic variability is suggested to generate said distinctions of pigment concentrations between carrots of the same cultivar.

### 4.5 Future perspectives

The standardisation of systematic image acquisition protocols by the scientific community must be established. This demonstrates that, together with image pre-processing, it strongly determines the final capabilities of pigment content prediction of the model, thus avoiding false positives or artefacts. This includes the use of a colour checker and gives way to assess the influence of different light conditions.

However, to strengthen the image analysis, it is recommended to directly measure the resulting light intensity that reaches the sample surface. Increases in sample size and genotypic diversity are proposed to make the model more robust. Similarly, exploring the effect of obtaining more colorimetric measurements per sample is suggested. The relationships between such variables may amplify the effect of individual carotenoids and allow for a greater understanding of the varying genetic expression of the pigments and the influence(s) of the environment – a topic that could be explored with mechanistic models. This information may aid breeding programmes in search of higher concentrations of the pigments.

A novelty of this study, however, is the exclusion of environmental variability in carrots and their carotenoid and anthocyanin combinations for the first time. A broader approach based on multiple species and their interactions with their environment could be explored in this sense. The expansion of such statistical models (e.g., to other food products) and subsequent development of colour-pigment content relationship databases may train artificial intelligence methods to develop robust automatic detection systems – the apparent basis for future agricultural and food technologies. This method could be useful when applied in phone applications (together with AI) for simple detections of pigment content, and therefore the antioxidant capacity, of fresh produce. For instance, the establishment of claims of the provitamin/antioxidant capacity of carotenoids and anthocyanins could be based on this method. Therefore, retailers and hospitals could reduce waste by selecting food products in an accessible and well-informed manner.

## 5 Conclusion

The current study was designed with the aim of robustly exploring the colour-pigment relationships of carotenoids and anthocyanins in fresh carrots of different colours via digital image colour analysis under six different light conditions. Colour-pigment relationships are traditionally assessed with rudimentary linear regressions in the literature, inappropriately disregarding the multicollinearity of colour parameters in multi-dimensional colour spaces (e.g., CIELab/Ch). Additionally, the light environment during image acquisition was found to influence the weight of each colour parameter later during statistical analysis. Employing partial least squares regression accounted for the multicollinearity of colour parameters and successfully predicted total carotenoid and total anthocyanin content in a stable manner, especially when using the *WF 0* and *PF 0* light conditions. The final model (*WF 0*) was selected for best fit and its close similarity to a default digital camera and lighting set-up (highly accessible), thus offering a cost-effective alternative to traditional laboratory methods.

The current study suggests a detailed protocol for rapid, non-destructive assessment of total carotenoid and anthocyanin contents with aims to standardise image phenotyping methods and further inform publicly accessible data on genetically diverse carrot cultivars (resulting in different root colours). This information can be used to aid breeding programmes in search of higher concentrations of these powerful antioxidants and determinants of produce colour and quality.

**Non-standard abbreviations**.

The following is a list of abbreviations used in the manuscript that are not considered standard or widely recognised in the field. Image analysis optimisation (IAO), orange carrot cultivars (O1–O9), purple carrot cultivars (P1–P2), polarising filter (PF), purple-yellow carrot cultivar (PY1), red carrot cultivar (R1), total anthocyanin content (TAC), total carotenoid content (TCC), white carrot cultivar (W1), without filter (WF), yellow carrot cultivars (Y1–Y2).

## Data Availability

Data and protocols are available in the following links: https://doi.org/10.34894/P37WCL, https://doi.org/10.34894/OUURRH, https://doi.org/10.34894/BTPTSV.

## Supplementary material

Supplementary Figure 1 **(A**) No selection; **(B)** automatic lasso; and **(C)** selection by hand of the image analysis of two carrot disks (Carrot 1 and Carrot 2) to assess the accuracy of each selection method. Automatic lasso included sections of peel (unwanted) and omitted parts of the front face flesh (wanted), while selection by hand allowed precision to include only the wanted flesh into the image analysis.

Supplementary Figure 2 **(A)** Purple cultivar (P1); **(B)** orange cultivar (O5); and **(C)** white cultivar (W1) carrot disks imaged under the polarised filter (*PF 0)* and the default, without filter (*WF 0)*, light conditions showing the differences in both aperture (expressed in f-number; f/#) and exposure time (expressed in seconds, e.g., 1/80s). The automatic response of the digital camera to genotypically diverse sample disks is not consistent between the two light conditions nor within each cultivar.

Supplementary Figure 3 Scatterplots visualising the actual pigment content values against the values predicted by four partial least squares (PLS) models under the following circumstances: **(A)** total carotenoid content (TCC) estimation under default, *WF 0*, light condition; **(B)** TCC estimation under polarised *PF 0* light condition; **(C)** total anthocyanin content (TAC) estimation under default *WF 0* light condition; and **(C)** TAC estimation under polarised *PF 0* light condition.
