# Supplementary figures and images for: Image analysis optimisation for carotenoid and anthocyanin content prediction in carrots: addressing colour parameter multicollinearity and genotypic diversity
Source: Front Plant Sci. 2026 May 20;17:1713048. doi: 10.3389/fpls.2026.1713048 (PMC13229846; doi:10.3389/fpls.2026.1713048)

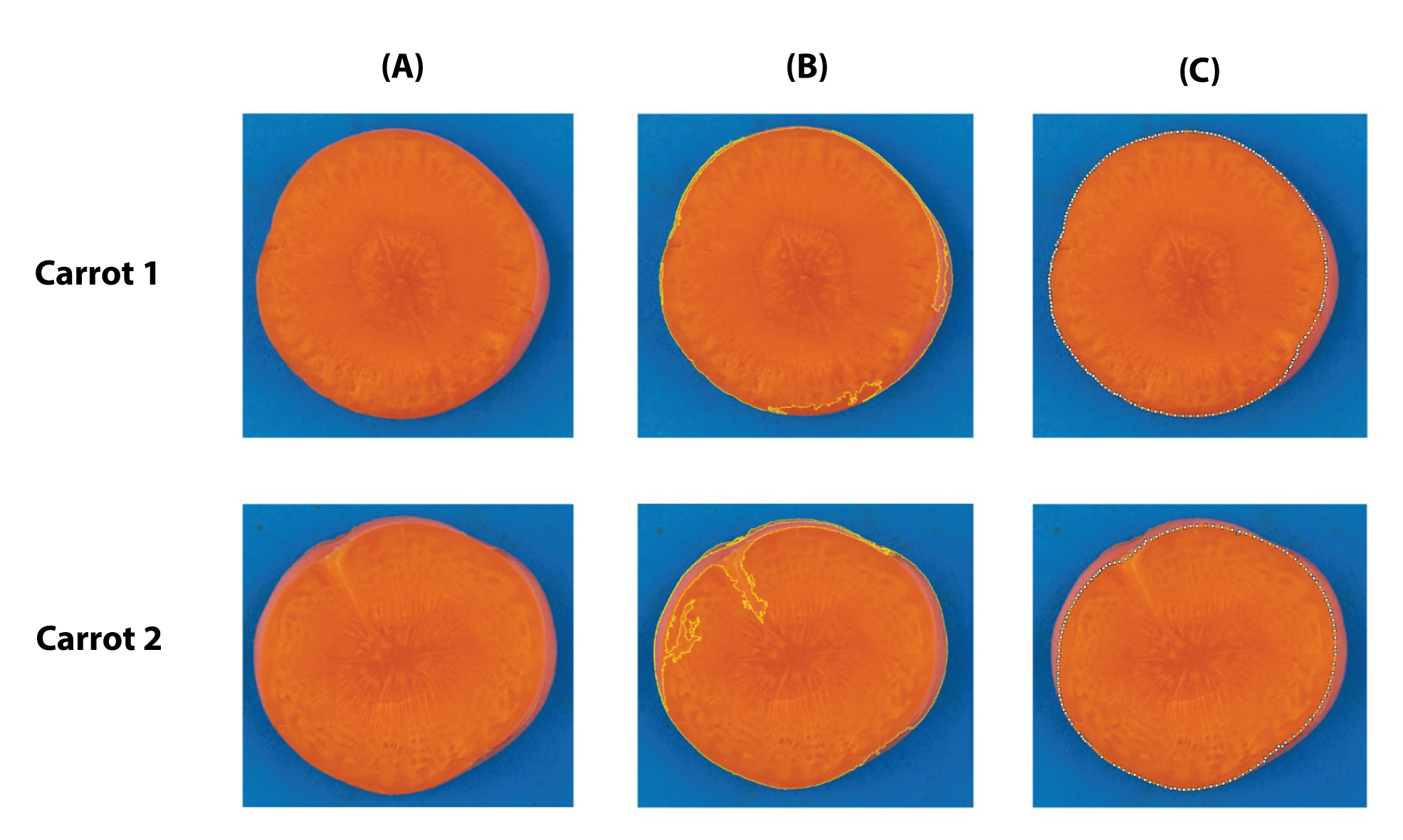

Supplement: Supplementary Figure 1 — (A) No selection; (B) automatic lasso; and (C) selection by hand of the image analysis of two carrot disks (Carrot 1 and Carrot 2) to assess the accuracy of each selection method. Automatic lasso included sections of peel (unwanted) and omitted parts of the front face flesh (wanted), while selection by hand allowed precision to include only the wanted flesh into the image analysis. [file Image1.tif]

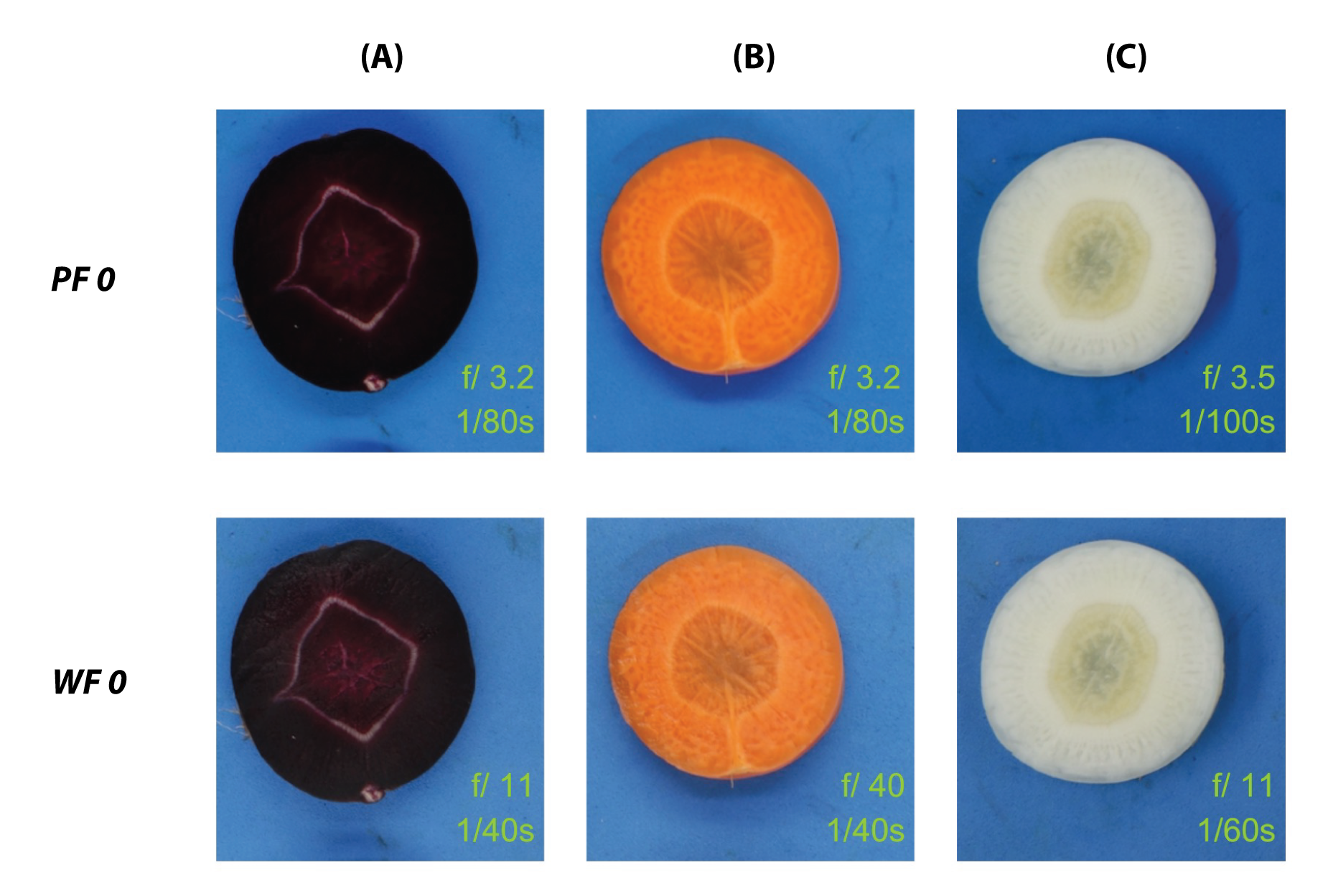

Supplement: Supplementary Figure 2 — (A) Purple cultivar (P1); (B) orange cultivar (O5); and (C) white cultivar (W1) carrot disks imaged under the polarised filter (PF 0) and the default, without filter (WF 0), light conditions showing the differences in both aperture (expressed in f-number; f/#) and exposure time (expressed in seconds, e.g., 1/80s). The automatic response of the digital camera to genotypically diverse sample disks is not consistent between the two light conditions nor within each cultivar. [file Image2.tif]

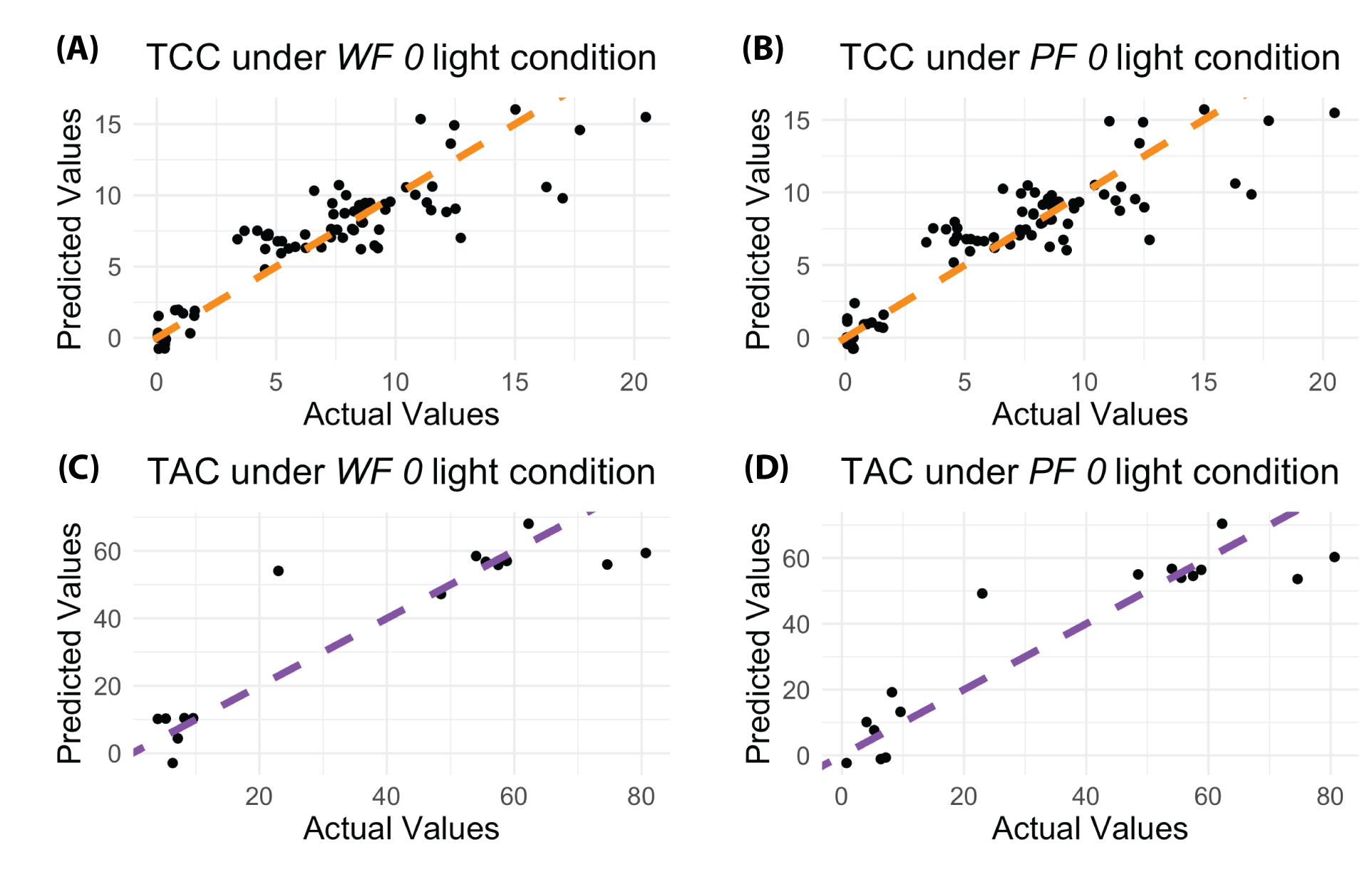

Supplement: Supplementary Figure 3 — Scatterplots visualising the actual pigment content values against the values predicted by four partial least squares (PLS) models under the following circumstances: (A) total carotenoid content (TCC) estimation under default, WF 0, light condition; (B) TCC estimation under polarised PF 0 light condition; (C) total anthocyanin content (TAC) estimation under default WF 0 light condition; and (C) TAC estimation under polarised PF 0 light condition. [file Image3.tif]
